# Supplementary material for: A Microbiological Approach to Alleviate Soil Replant Syndrome in Peaches
Source: Microorganisms. 2023 May 30;11(6):1448. doi: 10.3390/microorganisms11061448 (PMC10303857; doi:10.3390/microorganisms11061448)
Supplement: Supplementary file 1 [file microorganisms-11-01448-s001.zip › microorganisms-2381389-supplementary.pdf]

Supplementary tables and figures:

Supplementary Table S1. Soil Analysis of autoclaved and non-autoclaved soils by treatment. Each treatment was a subset and consisted of n=3 replicates. Mean and standard deviation were calculated using Microsoft Excel.

Treatment	Peach Dry Total Biomass (g)	1:1 Soil pH	Organic Matter %	H2O Total N ppm	Organic N ppm	H3A Nitrate ppm	Ammonium ppm	H3A Inorganic Nitrogen ppm	H3A Total Phosphorus	Inorganic Phosphorus
Alfalfa NonAutoclaved (Mean ± STD)	4.02 ± 1.24	8.13 ± 0.25	1.8 ± 0.1	15.1 ± 2.23	9.77 ± 0.45	4.23 ± 1.63	0.7 ± 0.1	4.93 ± 1.71	4.67 ± 0.58	2.8 ± 0.26
Fescue NonAutoclaved (Mean ± STD)	2.83 ± 0.33	8.07 ± 0.12	1.77 ± 0.06	11.9 ± 2	7.93 ± 1.45	3.2 ± 0.66	0.8 ± 0.1	4 ± 0.56	6 ± 1	3.87 ± 0.75
Corn NonAutoclaved (Mean ± STD)	2.87 ± 0.58	8.1 ± 0.1	1.63 ± 0.06	12.93 ± 1.52	9.13 ± 1.64	3.17 ± 0.64	0.7 ± 0.1	3.83 ± 0.61	8 ± 2.65	5.23 ± 1.6
Tomato NonAutoclaved (Mean ± STD)	4.16 ± 0.28	8.1 ± 0.1	1.63 ± 0.06	13.8 ± 1.68	10.57 ± 1.05	2.8 ± 0.53	0.63 ± 0.06	3.43 ± 0.59	9.67 ± 0.58	6.37 ± 0.59
None NonAutoclaved (Mean ± STD)	2.37 ± 0.99	8.23 ± 0.06	1.5 ± 0	13.03 ± 0.25	8.83 ± 1.03	3.4 ± 0.46	0.37 ± 0.31	3.8 ± 0.53	10.33 ± 1.15	7.2 ± 1.21
Alfalfa Autoclaved (Mean ± STD)	1.71 ± 1.16	8.07 ± 0.06	1.7 ± 0.1	14.87 ± 1.42	7.4 ± 0.82	6.07 ± 1.68	1 ± 0.2	7.03 ± 1.72	6.33 ± 1.15	4.07 ± 0.84
Fescue Autoclaved (Mean ± STD)	0.72 ± 0.12	8.07 ± 0.06	1.6 ± 0.1	9.77 ± 1.59	6.97 ± 1.27	2.2 ± 1.91	1.17 ± 0.4	3.33 ± 1.81	4.33 ± 0.58	2.8 ± 0.44
Corn Autoclaved (Mean ± STD)	1.16 ± 0.59	8.1 ± 0.1	1.63 ± 0.12	10.5 ± 0.82	8.33 ± 0.96	1.63 ± 0.25	0.97 ± 0.47	2.6 ± 0.46	9 ± 4.58	6.43 ± 3.35
Tomato Autoclaved (Mean ± STD)	2.41 ± 0.28	8.13 ± 0.06	1.73 ± 0.12	14.7 ± 2.76	12.07 ± 0.76	2.27 ± 1.59	0.8 ± 0.1	3.07 ± 1.5	9 ± 1.73	6.17 ± 1.46
None Autoclaved (Mean ± STD)	2.75 ± 1.08	8.17 ± 0.06	1.57 ± 0.06	10.8 ± 0.95	8.87 ± 1.83	1.43 ± 0.15	0.87 ± 0.25	2.3 ± 0.1	11.67 ± 0.58	8.47 ± 0.35

Treatment	H3A Organic Phosphorus ppm	H3A ICAP Potassium ppm	Organic N:Inorganic N	Available N ppm	Available P ppm	Available K ppm	Total N Concentration %	Total P Concentration %	Total K Concentration %	CO2-C ppm
Alfalfa NonAutoclaved (Mean ± STD)	1.93 ± 0.38	90 ± 4	2.13 ± 0.67	22.03 ± 2.63	10.9 ± 1.35	4.71	0.09 ± 0.02	0.06 ± 0	0.22 ± 0.01	36.6 ± 11.70
Fescue NonAutoclaved (Mean ± STD)	2 ± 0.2	89 ± 4.58	2 ± 0.36	17.93 ± 2.32	13.43 ± 2.15	5.34	0.08 ± 0.01	0.06 ± 0	0.23 ± 0.01	26.67 ± 2.55
Corn NonAutoclaved (Mean ± STD)	2.77 ± 0.81	91 ± 5.57	2.43 ± 0.67	19.43 ± 2.34	18.2 ± 5.07	7.09	0.09 ± 0.01	0.06 ± 0	0.24 ± 0.01	26.2 ± 6.24
Tomato NonAutoclaved (Mean ± STD)	3 ± 0.3	83.67 ± 4.62	3.17 ± 0.55	21.03 ± 1.93	20.7 ± 1.8	5.29	0.08 ± 0	0.06 ± 0	0.23 ± 0.01	24.63 ± 0.67
None NonAutoclaved (Mean ± STD)	2.87 ± 0.21	89.67 ± 4.93	2.37 ± 0.57	17.6 ± 1.54	21.03 ± 2.47	107.17 ±	0.08 ± 0.02	0.06 ± 0	0.23 ± 0.01	18.17 ± 1.98
Alfalfa Autoclaved (Mean ± STD)	2.03 ± 0.55	74 ± 4.36	1.07 ± 0.25	21.7 ± 3.3	14 ± 3.3	88.9 ± 4.69	0.08 ± 0.01	0.06 ± 0.01	0.22 ± 0.02	26 ± 7.65
Fescue Autoclaved (Mean ± STD)	1.53 ± 0.4	81.67 ± 1.15	2.57 ± 1.43	14.2 ± 2.49	9.47 ± 2.53	97.8 ± 1.91	0.08 ± 0.01	0.06 ± 0	0.22 ± 0.01	21.13 ± 8.96
Corn Autoclaved (Mean ± STD)	2.6 ± 0.96	87.67 ± 9.61	3.3 ± 0.46	15.23 ± 2.85	18.97 ± 8.5	11.94	0.11 ± 0.05	0.05 ± 0.02	0.15 ± 0.1	20.17 ± 3.45
Tomato Autoclaved (Mean ± STD)	2.83 ± 0.35	82 ± 12.49	4.5 ± 1.59	22.7 ± 3.08	20.47 ± 4.05	98.43 ±	0.08 ± 0.01	0.06 ± 0	0.25 ± 0.02	37.8 ± 10.90
None Autoclaved (Mean ± STD)	3.23 ± 0.15	81.67 ± 2.08	3.87 ± 0.97	16.23 ± 2.14	25.43 ± 1.4	97.57 ±	0.07 ± 0	0.06 ± 0	0.23 ± 0.01	25.4 ± 7.73

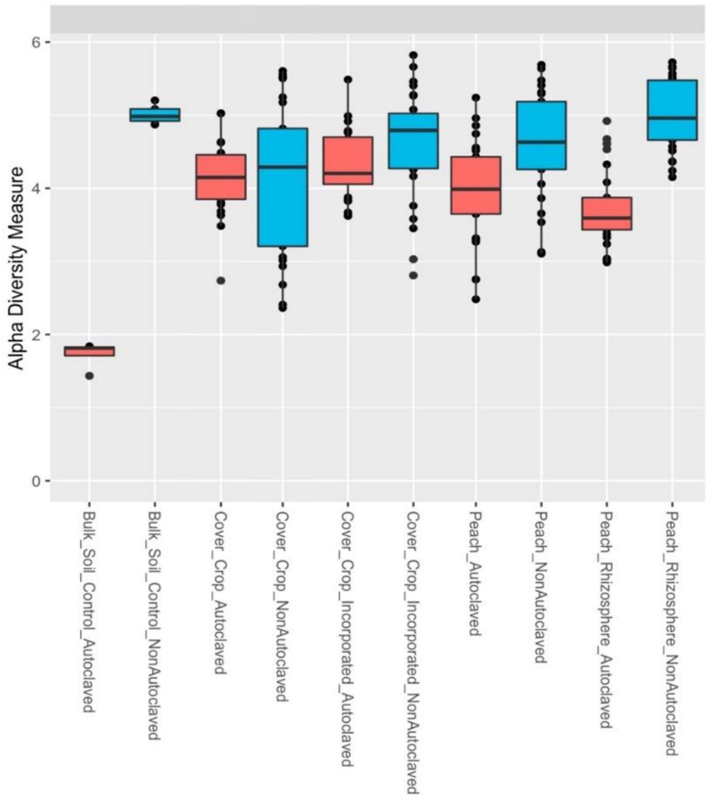

Supplementary Figure S1. Shannon index of controls and all treatments separated by autoclaved (red), non-autoclaved (blue), and cover crop history.

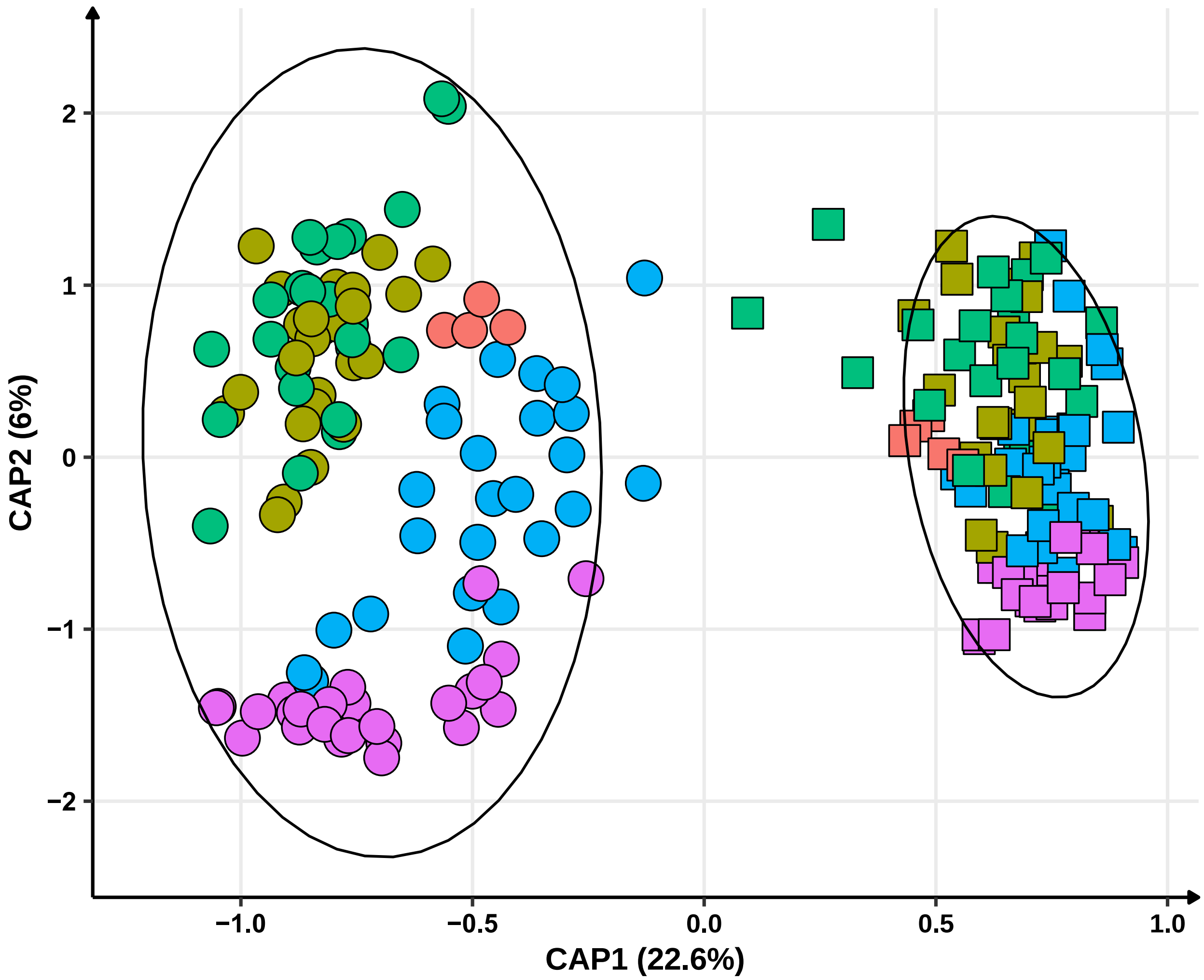

Supplementary Figure S2. Principal Coordinate Analysis (PCoA) of bacterial DNA using Bray-Curtis distance for bacterial microbiomes of all samples sorted by crop cycle. Shape denotes soil treatment with circles representing autoclaved and squares non-autoclaved. Color indicated crop cycle with initial bulk soil control (before any crops) in salmon, cover crop bulk soil in olive green, incorporated cover crop bulk soil in leaf green, peach bulk soil in blue, and peach rhizosphere in purple.

Supplementary Table S2. Differential abundance of bacterial taxa with increased abundance in autoclaved cover crop bulk soil as compared to non-autoclaved cover crop bulk soil. P-adjusted values which are <0.001 are represented as 0.000. Log2 Fold change was based on log2FC=Log2(non-autoclaved)-Log2(autoclaved) using the DESeq2 package in RStudio.

	**Alfalfa**		**Fescue**		**Corn**		**Tomato**		**No Crop**		
Bacterial Taxa	Log2FC	Padjust	Log2 Fold	Padjust	Log2	Padjust	Log2	Padjust	Log2	Padjust	Count
Adhaeribacter aquaticus	-8.315	0.000	-7.528	0.003	-7.560	0.016	-6.844	0.003	-7.791	0.001	5
Ammoniphilus resinae	-4.088	0.021	-4.977	0.003	-4.898	0.005	-3.487	0.006	-3.780	0.008	5
Cytobacillus firmus	-7.664	0.002	-7.795	0.000	-6.919	0.003	-8.147	0.000	-8.027	0.000	5
Cytobacillus oceanisediminis	-5.144	0.000	-5.811	0.000	-4.246	0.000	-4.406	0.000	-5.055	0.000	5
Fictibacillus arsenicus	-4.760	0.033	-5.943	0.000	-4.564	0.010	-3.500	0.002	-5.014	0.000	5
Fictibacillus phosphorivorans	-5.646	0.005	-7.064	0.000	-5.501	0.003	-4.544	0.000	-5.356	0.000	5
Mesobacillus subterraneus	-5.006	0.000	-6.017	0.000	-2.923	0.014	-3.325	0.000	-3.793	0.000	5
Planomicrobium chinense	-7.670	0.007	-9.049	0.000	-22.529	0.000	-6.451	0.012	-8.814	0.000	5
Planomicrobium glaciei	-9.974	0.000	-12.347	0.000	-7.860	0.000	-9.158	0.000	-9.662	0.000	5
Pontibacter brevis	-8.026	0.000	-8.628	0.000	-6.958	0.015	-8.983	0.000	-6.350	0.017	5
Pontibacter chitinilyticus	-7.224	0.005	-8.455	0.001	-7.023	0.014	-8.278	0.000	-6.436	0.016	5
Pontibacter korlensis	-9.310	0.000	-10.047	0.000	-8.007	0.006	-9.299	0.000	-6.923	0.001	5
Pontibacter populi	-12.511	0.000	-11.646	0.000	-10.162	0.000	-11.068	0.000	-8.624	0.000	5
Tumebacillus soli	-4.962	0.023	-6.566	0.000	-6.988	0.000	-4.569	0.000	-5.400	0.000	5
Ammoniphilus oxalaticus	-7.422	0.022	-7.543	0.001			-4.028	0.050	-5.451	0.015	4
Bacillus onubensis	-6.307	0.032	-7.211	0.000			-7.280	0.000	-7.107	0.000	4
Telluribacter humicola	-7.308	0.025	-9.784	0.000			-8.772	0.000	-8.715	0.000	4
Leptolyngbya sp. NIES-3755	-23.926	0.000	-8.488	0.001	-7.594	0.004			-5.708	0.031	4
Asprobacter aquaticus			-6.984	0.022	-6.182	0.016	-6.704	0.003	-5.028	0.011	4
Bacillus dafuensis			-5.182	0.002	-5.320	0.005	-4.363	0.006	-4.753	0.002	4
Bacillus humi			-6.042	0.000	-5.140	0.017	-4.208	0.004	-4.489	0.017	4
Bacillus infantis			-4.786	0.004	-4.110	0.032	-2.915	0.021	-3.826	0.007	4
Chungangia koreensis			-7.501	0.001	-5.877	0.041	-7.746	0.000	-6.260	0.004	4
Exiguobacterium sibiricum			-9.845	0.000	-24.061	0.000	-23.836	0.000	-7.022	0.001	4

Flavihumibacter cheonanensis			-8.992	0.000	-7.505	0.001	-11.570	0.000	-11.534	0.000	4
Flavihumibacter stibioxidans			-7.354	0.003	-6.305	0.007	-9.672	0.000	-9.517	0.000	4
Massilia armeniaca			-5.858	0.033	-6.385	0.043	-6.462	0.011	-6.363	0.007	4
Flavihumibacter petaseus	-6.839	0.036					-7.313	0.001	-7.193	0.000	3
Reyranella massiliensis	-6.467	0.028	-5.668	0.022					-5.351	0.006	3
Planococcus plakortidis	-6.806	0.020	-6.149	0.007					-5.602	0.014	3
Pontibacter rhizosphera	-7.883	0.006	-7.985	0.000			-6.821	0.007			3
Adhaeribacter aerolatus			-8.003	0.000			-6.731	0.001	-7.320	0.002	3
Alkalihalobacillus halodurans			-5.527	0.015	-6.537	0.011			-3.692	0.042	3
Bacillus methanolicus			-4.580	0.008			-2.744	0.044	-3.014	0.041	3
Cytobacillus gottheilii			-4.512	0.010			-3.065	0.013	-3.351	0.029	3
Daejeonella rubra			-7.355	0.003			-5.927	0.019	-7.643	0.000	3
Domibacillus tundrae			-4.498	0.046			-4.627	0.047	-5.228	0.029	3
Exiguobacterium mexicanum			-23.542	0.000			-9.426	0.000	-25.972	0.000	3
Exiguobacterium sp. MH3			-10.485	0.000	-24.982	0.000			-7.650	0.001	3
Exiguobacterium sp. U13-1			-9.765	0.000	-23.867	0.000			-7.249	0.002	3
Exiguobacterium sp. ZWU0009			-8.400	0.000	-7.092	0.024			-5.879	0.026	3
Flavihumibacter profundi			-6.740	0.014			-9.264	0.000	-8.827	0.000	3
Flavisolibacter sp. 17J28-1			-8.994	0.000			-5.476	0.000	-7.911	0.000	3
Noviherbaspirillum aurantiacum			-7.210	0.008	-7.304	0.011	-8.069	0.000			3
Paenibacillus yunnanensis			-6.498	0.003			-6.324	0.012	-6.639	0.005	3
Pseudomonas stutzeri			-5.964	0.003	-5.525	0.045			-9.264	0.000	3
Pseudorhodoferax soli			-6.626	0.016	-6.187	0.030	-5.754	0.040			3
Shinella sp. HZN7			-6.256	0.022			-6.381	0.021	-6.853	0.001	3
Tumebacillus algifaecis			-6.513	0.002	-7.540	0.000	-5.087	0.009			3
Tumebacillus flagellatus			-4.563	0.022	-6.563	0.000	-4.004	0.028			3
Tumebacillus ginsengisoli			-3.864	0.017	-3.962	0.000	-2.767	0.015			3

Georhizobium profundi					-6.919	0.009	-6.644	0.018	-7.101	0.001	3
Tumebacillus lipolyticus					-7.459	0.010	-7.543	0.001	-5.362	0.042	3
Adhaeribacter swui	-6.452	0.030							-5.953	0.025	2
Hyphomonas neptunium	-23.038	0.000							-7.363	0.000	2
Methylovorus glucosotrophus	-6.633	0.026							-5.982	0.025	2
Symbiobacterium thermophilum	-6.972	0.007							-6.631	0.005	2
Lysobacter oligotrophicus	-9.119	0.001			-8.316	0.004					2
Leptolyngbya boryana	-6.116	0.034			-4.935	0.029					2
Acaryochloris marina	-7.613	0.020	-6.653	0.007							2
Achromobacter spanius			-6.925	0.005					-9.262	0.000	2
Actinotalea ferrariae			-6.525	0.033					-5.570	0.037	2
Bacillus mediterraneensis			-5.241	0.005					-4.312	0.014	2
Devosia ginsengisoli			-6.073	0.014					-6.819	0.001	2
Devosia soli			-6.310	0.005					-6.547	0.013	2
Dyadobacter fermentans			-6.979	0.012					-5.779	0.029	2
Dyadobacter jiangsuensis			-7.245	0.001					-6.609	0.005	2
Exiguobacterium acetylicum			-10.469	0.000					-7.661	0.001	2
Flavisolibacter galbus			-7.452	0.001			-5.949	0.033			2
Flavisolibacter ginsengiterrae			-5.694	0.000					-2.720	0.040	2
Larkinella harenae			-5.476	0.046			-6.580	0.001			2
Noviherbaspirillum soli			-7.908	0.000			-3.964	0.030			2
Novosphingobium aromaticivorans			-6.621	0.029			-6.943	0.012			2
Planococcus rifietoensis			-4.827	0.036					-4.136	0.037	2
Pontibacter ruber			-5.513	0.046			-5.915	0.020			2
Shinella zoogloeoides			-6.923	0.005					-6.398	0.003	2
Azospirillum sp. TSH58			-6.822	0.006	-7.515	0.004					2
Oxalophagus oxalicus			-5.434	0.008	-5.078	0.019					2
Mesorhizobium sp. Pch-S					-7.132	0.006	-5.128	0.041			2
Nostoc sphaeroides					-22.872	0.000	-6.550	0.006			2

Pseudomonas sp. UW4					-6.214	0.048			-7.022	0.008	2
Aneurinibacillus migulanus							-6.362	0.022	-5.913	0.026	2
Aneurinibacillus soli							-6.325	0.012	-5.962	0.025	2
Aneurinibacillus tyrosinisolvens							-5.732	0.040	-6.318	0.017	2
Flavihumibacter sediminis							-6.069	0.030	-6.599	0.005	2
Hydrogenophaga atypica							-6.193	0.014	-5.752	0.029	2
Hydrogenophaga sp. PBC							-7.087	0.002	-10.846	0.000	2
Larkinella rosea							-6.818	0.003	-6.085	0.024	2
Methylobacillus flagellatus							-6.451	0.020	-6.817	0.004	2
Quasibacillus thermotolerans							-4.204	0.011	-3.187	0.050	2
Roseomonas aestuarii							-6.522	0.019	-6.137	0.020	2
Sinorhizobium sp. RAC02							-5.762	0.040	-6.318	0.003	2
Agaricicola taiwanensis	-22.968	0.000									1
Fischerella sp. NIES-3754	-23.771	0.000									1
Thermincola potens	-23.587	0.000									1
Alkalihalobacillus pseudofirmus			-7.491	0.000							1
Arenimonas daechungensis			-6.514	0.018							1
Arthrobacter sp. PGP41			-4.610	0.045							1
Azospirillum sp. TSA2s			-9.475	0.000							1
Bacillus dakarensis			-5.444	0.029							1
Bacillus mannanilyticus			-6.007	0.017							1
Exiguobacterium antarcticum			-6.865	0.005							1
Flaviaesturariibacter luteus			-7.523	0.006							1
Flavisolibacter ginsengisoli			-8.113	0.000							1
Flavisolibacter ginsenosidimutans			-3.263	0.036							1
Kocuria rosea			-6.861	0.025							1
Lysobacter brunescens			-6.789	0.019							1
Lysobacter solanacearum			-21.908	0.000							1

Magnetospirillum bellicus	-6.539	0.033							1
Neobacillus notoginsengisoli	-5.407	0.049							1
Niastella hibisci	-6.978	0.011							1
Nocardioides sp. JS614	-6.909	0.022							1
Paenarthrobacter nicotinovorans	-6.533	0.033							1
Paenibacillus chitinolyticus	-4.521	0.046							1
Paenibacillus ihbetae	-6.691	0.006							1
Paenibacillus typhae	-5.215	0.044							1
Pantoea agglomerans	-21.945	0.000							1
Parasegetibacter luojiensis	-12.965	0.000							1
Planctopirus ephydatiae	-6.880	0.040							1
Planococcus kocurii	-5.967	0.049							1
Planococcus sp. MB-3u-03	-6.475	0.009							1
Pontibacter amylolyticus	-9.390	0.000							1
Pontibacter diazotrophicus	-6.586	0.017							1
Pontibacter virosus	-22.928	0.000							1
Pseudarthrobacter chlorophenolicus	-6.266	0.041							1
Pseudohongiella spirulinae	-5.612	0.041							1
Sphingomonas koreensis	-6.409	0.010							1
Verrucomicrobia bacterium IMCC26134	-6.846	0.006							1
Arthrobacter sp. Rue61a			-6.727	0.024					1
Arthrobacter sp. YN			-7.152	0.013					1
Brevundimonas diminuta			-6.190	0.030					1
Paenibacillus sp. FSL H7-0357			-6.457	0.043					1
Pelosinus fermentans			-6.431	0.043					1
Rhodococcus sp. NJ-530			-6.708	0.020					1
Verrucomicrobium spinosum			-7.915	0.003					1

Brevundimonas sp. MF30-B	-6.493	0.010			1
Chthoniobacter flavus	-2.447	0.032			1
Cnuella takakiae	-4.681	0.050			1
Gemmobacter megaterium	-6.589	0.017			1
Haematobacter massiliensis	-5.542	0.020			1
Janthinobacterium sp. Marseille	-5.865	0.036			1
Nostoc punctiforme	-6.139	0.010			1
Oligoflexus tunisiensis	-4.482	0.050			1
Phenylobacterium muchangponense	-5.534	0.049			1
Rhodobacter capsulatus	-5.991	0.021			1
Rhodobacter sphaeroides	-6.161	0.014			1
Rufibacter sp. DG31D	-5.656	0.045			1
Tabrizicola piscis	-4.264	0.031			1
Achromobacter insolitus			-6.961	0.003	1
Achromobacter xylosoxidans			-6.479	0.006	1
Acidovorax defluvii			-7.067	0.007	1
Acidovorax facilis			-7.654	0.003	1
Acidovorax sp. KKS102			-6.453	0.014	1
Acidovorax sp. RAC01			-6.945	0.008	1
Agrobacterium tumefaciens			-6.047	0.037	1
Algoriphagus jejuensis			-7.189	0.003	1
Algoriphagus terrigena			-6.885	0.018	1
Aminobacter aminovorans			-7.084	0.001	1
Bacillus tianmuensis			-5.467	0.024	1
Bacillus timonensis			-5.392	0.027	1
Brevundimonas sp. M20			-5.773	0.016	1
Cellvibrio sp. KY-YJ-3			-7.647	0.004	1
Cellvibrio sp. PSBB023			-6.612	0.013	1
Cohnella candidum			-3.785	0.030	1
Devosia geojensis			-5.405	0.025	1

Devosia riboflavina	-6.162	0.033	1
Devosia sp. A16	-3.361	0.025	1
Devosia sp. I507	-5.425	0.040	1
Ensifer adhaerens	-5.309	0.018	1
Exiguobacterium aurantiacum	-8.480	0.001	1
Exiguobacterium sp. AT1b	-6.012	0.039	1
Fermentibacillus polygoni	-5.326	0.043	1
Herbaspirillum seropedicae	-6.920	0.003	1
Hydrogenophaga sp. RAC07	-23.204	0.000	1
Janthinobacterium sp. 17J80-10	-5.966	0.039	1
Lacunisphaera limnophila	-5.744	0.031	1
Legionella massiliensis	-4.140	0.015	1
Legionella saoudiensis	-5.610	0.033	1
Luteolibacter flavescens	-4.399	0.029	1
Massilia oculi	-6.008	0.025	1
Massilia sp. WG5	-6.326	0.017	1
Methyloversatilis discipulorum	-6.392	0.044	1
Microbacterium sp. ABRD_28	-22.901	0.000	1
Nibricoccus aquaticus	-3.681	0.042	1
Paenibacillus koleovorans	-5.331	0.027	1
Pararcticibacter amylolyticus	-6.651	0.001	1
Phenylobacterium haematophilum	-5.979	0.013	1
Prosthecobacter dejongeii	-6.033	0.011	1
Prosthecobacter fusiformis	-7.013	0.003	1
Pseudomonas alcaliphila	-6.584	0.038	1
Pseudomonas koreensis	-6.104	0.012	1
Pseudomonas putida	-8.428	0.000	1
Pseudoxanthomonas mexicana	-8.210	0.000	1
Shinella yambaruensis	-5.918	0.014	1

Siccirubricoccus deserti									-6.011	0.013	1
Synechococcus elongatus									-22.702	0.000	1

Supplementary Table S3. Differential abundance of bacterial taxa with increased abundance in non-autoclaved cover crop bulk soil as compared to autoclaved cover crop bulk soil. P-adjusted values which are <0.001 are represented as 0.000. Log2 Fold change was based on log2FC=Log2(non-autoclaved)-Log2(autoclaved) using the DESeq2 package in RStudio.

	**Alfalfa**		**Fescue**		**Corn**		**Tomato**		**No Crop**		
Bacterial Taxa	Log2	Padjust	Log2	Padjust	Log2	Padjust	Log2	Padjust	Log2	Padjust	Count
[*Brevibacterium*] *frigoritolerans*	4.22	0.01	4.11	0.03	3.97	0.02	5.56	0.00	3.99	0.00	5
Aciditerrimonas ferrireducens	6.95	0.00	5.58	0.00	9.35	0.00	5.88	0.00	6.69	0.00	5
Bacillus megaterium	5.04	0.00	4.22	0.00	6.10	0.00	6.33	0.00	4.41	0.00	5
Baekduia soli	7.91	0.00	7.23	0.00	7.76	0.00	8.35	0.00	8.74	0.00	5
Brevitalea aridisoli	8.64	0.00	8.12	0.00	8.02	0.00	8.75	0.00	9.01	0.00	5
Brevitalea deliciosa	10.43	0.00	9.73	0.00	9.85	0.00	10.57	0.00	10.75	0.00	5
Gaiella occulta	9.89	0.00	9.35	0.00	9.43	0.00	10.15	0.00	10.33	0.00	5
Methyloceanibacter caenitepidi	7.96	0.00	7.78	0.00	7.77	0.00	8.02	0.00	8.34	0.00	5
Nitrospira japonica	9.12	0.00	8.32	0.00	8.58	0.00	9.20	0.00	9.82	0.00	5
Nostoc sp. HK-01	10.57	0.00	15.00	0.00	13.10	0.00	7.93	0.00	22.17	0.00	5
Nostoc sp. PCC 7107	24.47	0.00	14.16	0.00	12.07	0.00	6.93	0.01	22.68	0.00	5
Paenibacillus castaneae	8.19	0.00	5.31	0.02	7.58	0.00	8.26	0.00	5.76	0.00	5
Planctomycetes bacterium Pla175	8.05	0.00	4.64	0.02	7.87	0.00	8.56	0.00	8.60	0.00	5
Povalibacter uvarum	8.90	0.00	6.29	0.00	4.49	0.01	8.93	0.00	4.71	0.00	5
Rhodoplanes sp. Z2-YC6860	8.28	0.00	3.61	0.03	3.97	0.02	4.30	0.00	4.30	0.00	5
Skermanella mucosa	7.36	0.00	6.42	0.02	6.61	0.01	7.25	0.00	7.62	0.00	5
Skermanella rosea	6.63	0.00	8.86	0.00	9.30	0.00	9.38	0.00	9.37	0.00	5
Skermanella stibiiresistens	10.48	0.00	10.01	0.00	10.29	0.00	10.43	0.00	10.55	0.00	5
Solirubrobacter ginsenosidimutans	7.22	0.00	6.19	0.02	6.63	0.00	7.16	0.00	7.44	0.00	5
Solirubrobacter soli	8.10	0.00	5.25	0.02	7.53	0.00	8.49	0.00	9.00	0.00	5
Sphingomonas daechungensis	7.65	0.00	6.51	0.02	7.26	0.00	7.55	0.00	8.22	0.00	5
Sphingomonas sediminicola	6.40	0.02	6.06	0.04	6.53	0.01	6.71	0.00	7.23	0.00	5
Sphingomonas sp. AE3	7.06	0.00	6.17	0.02	7.12	0.00	6.34	0.00	6.51	0.01	5
Steroidobacter denitrificans	5.33	0.00	4.92	0.00	4.11	0.01	5.94	0.00	3.96	0.00	5
Vicinamibacter silvestris	4.26	0.00	3.42	0.03	3.64	0.00	4.12	0.00	4.03	0.00	5
Woeseia oceani	7.08	0.00	6.72	0.01	6.91	0.00	7.62	0.00	7.74	0.00	5

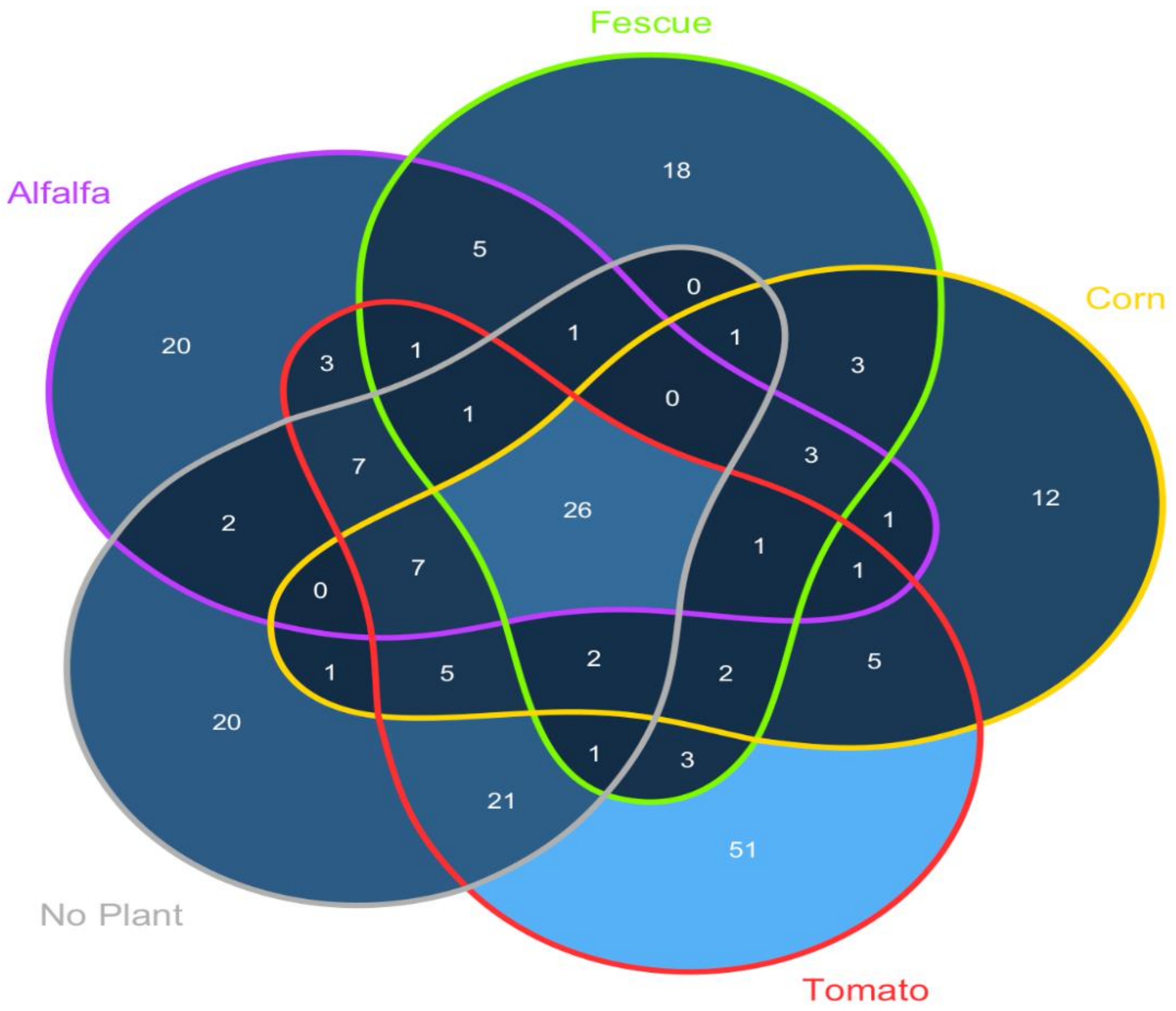

Supplementary Figure S3. Differential abundance Venn diagram of non-autoclaved cover crop bulk soils as compared to autoclaved cover crop bulk soils separated by crop. Orange circle indicates common bacteria found to be promoted within the 4 cover crops (alfalfa, fescue, corn, and tomato) treatments. A blue color gradient displays low bacterial taxa species counts in dark blue, with higher bacterial taxa species counts in lighter blue.

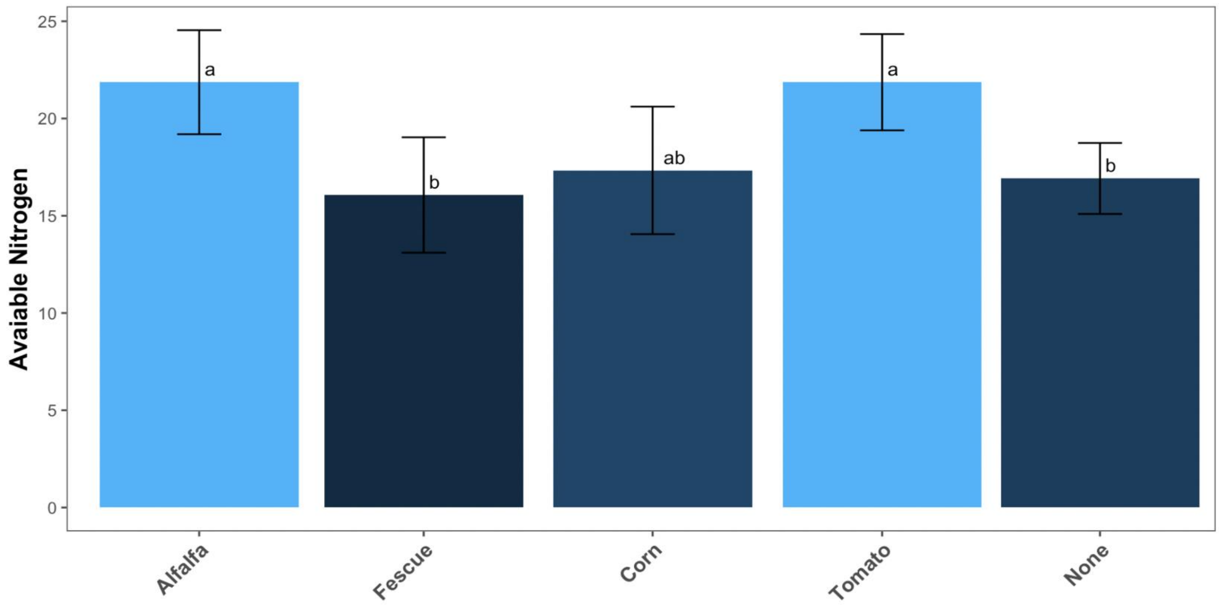

Supplementary Figure S4. Available organic nitrogen in correlation with total dry peach biomass. Of the samples with the highest available nitrogen, the top eight soil samples previously had either alfalfa or tomato from both soil treatment types (autoclaved / non-autoclaved). Different superscript letters denote significant difference (P < 0.01) compared with different cover crop histories.

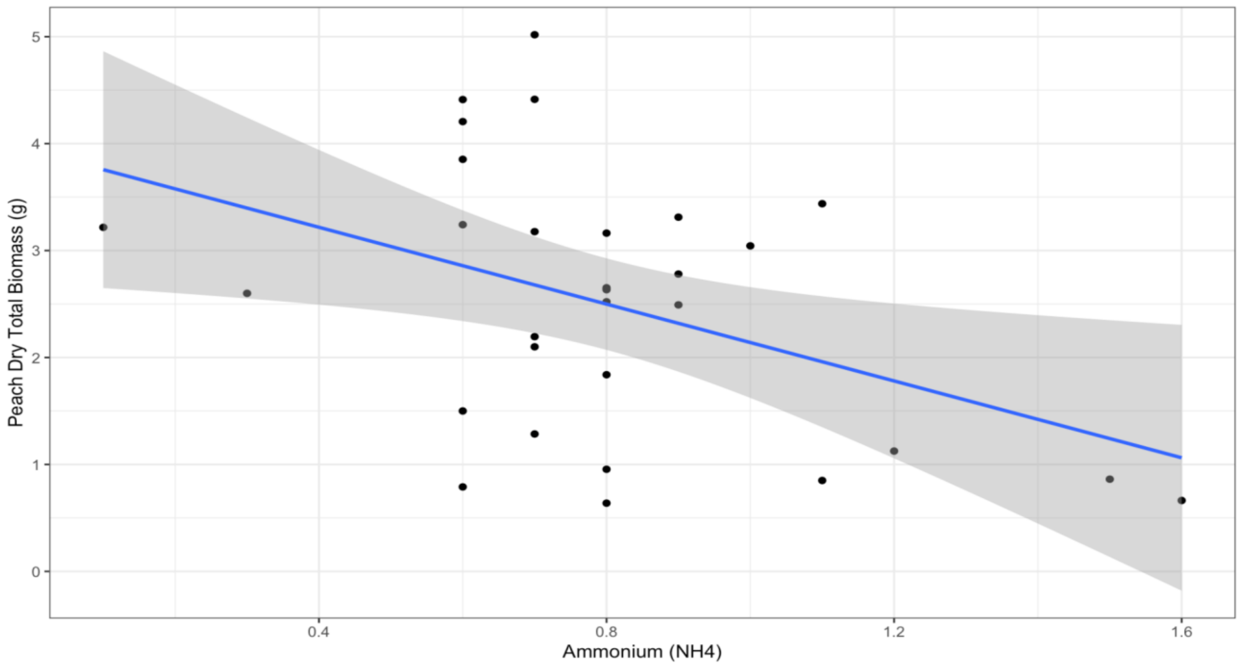

Supplementary Figure S5. Ammonium (NH_4) in correlation with total dry peach biomass. soil extractant (H3A-1) Correlation was positive (R^2= 0.1852, p-Value= 0.01761). Of the samples with the highest ammonium, previously disinfected soils showed consistently higher ammonium values.

Supplementary Table S4. Differential abundance of bacterial taxa with increased abundance in non-autoclaved peach bulk soil as compared to autoclaved peach bulk soil. P-adjusted values which are <0.001 are represented as 0.000. Log2 Fold change was based on log2FC=Log2(non-autoclaved)-Log2(autoclaved) using the DESeq2 package in RStudio.

	**Alfalfa**		**Fescue**		**Corn**		**Tomato**		**No Crop**		
Bacterial Taxa	Log2FC	Padjust	Log2 Fold	Padjust	Log2	Padjust	Log2	Padjust	Log2	Padjust	Count
Bacillus megaterium	*5.377*	0.037	4.575	0.041	5.405	0.011	6.885	0.009	4.374	0.009	5
Brevitalea aridisoli	*7.797*	0.015	8.441	0.001	8.816	0.000	8.672	0.001	8.161	0.000	5
Brevitalea deliciosa	*9.905*	0.000	10.134	0.000	7.045	0.007	10.251	0.000	9.821	0.000	5
Gaiella occulta	*8.641*	0.001	9.749	0.000	10.088	0.000	9.882	0.000	6.480	0.005	5
Nitrospira japonica	*8.427*	0.007	8.479	0.001	9.250	0.000	9.047	0.000	8.857	0.000	5
Skermanella rosea	*9.464*	0.000	8.958	0.000	5.603	0.038	9.068	0.000	9.120	0.000	5
Skermanella stibiiresistens	*10.49*	0.000	10.010	0.000	10.585	0.000	10.348	0.000	10.235	0.000	5
Sphingomonas daechungensis	*7.679*	0.018	7.445	0.015	7.839	0.010			7.812	0.005	4
Pelobacter acetylenicus	*7.459*	0.018	8.291	0.007	7.821	0.010			7.256	0.021	4
Methyloceanibacter caenitepidi			8.223	0.007	8.453	0.001	8.131	0.002	8.261	0.000	4
Paenibacillus castaneae			7.562	0.012	8.276	0.007	8.116	0.008	8.010	0.004	4
Woeseia oceani			7.565	0.012	7.961	0.009	7.652	0.003	6.834	0.029	4
[*Brevibacterium*] *frigoritolerans*	*8.020*	0.003					5.903	0.023	4.694	0.027	3
Peribacillus simplex	*7.506*	0.018	8.187	0.007			8.242	0.008			3
Longilinea arvoryzae	*5.946*	0.031	5.366	0.047					7.466	0.020	3
[*Desulfobacterium*] *catecholicum*	*8.843*	0.001	9.651	0.000	9.146	0.003					3
Gemmata sp. SH-PL17			8.450	0.001			8.296	0.001	8.002	0.004	3
Geoalkalibacter subterraneus			7.292	0.016			6.912	0.026	6.426	0.039	3
Arenimicrobium luteum					7.477	0.014	7.120	0.022	6.451	0.039	3
Skermanella aerolata	*8.538*	0.001					7.623	0.004			2

Solirubrobacter soli	*7.638*	0.018					8.358	0.001			2
Skermanella mucosa	*7.637*	0.006					7.077	0.023			2
Stigmatella aurantiaca	*6.994*	0.035			7.263	0.018					2
Thermomarinilinea lacunifontana			7.382	0.038					6.474	0.039	2
Desulforhopalus singaporensis			7.276	0.018	6.867	0.030					2
Bellilinea caldifistulae			6.986	0.049	7.484	0.014					2
Acidimicrobium ferrooxidans					7.431	0.015			7.036	0.011	2
Aridibacter nitratireducens					9.460	0.007			7.764	0.005	2
Burkholderiales bacterium GJ-E10					7.065	0.021			6.443	0.039	2
Planctomycetes bacterium Pla175					5.722	0.029			4.844	0.039	2
Gemmata obscuriglobus					7.254	0.018	6.675	0.034			2
Actinobacteria bacterium IMCC26256							7.019	0.023	6.368	0.040	2
Anaeromyxobacter sp. K							7.535	0.014	6.672	0.035	2
Bacillus safensis							7.281	0.019	6.223	0.045	2
Bacillus thuringiensis	*7.914*	0.014									1
Clostridium magnum	*9.325*	0.004									1
Haliscomenobacter hydrossis	*8.035*	0.035									1
Clostridium saccharoperbutylacetonicum			5.523	0.049							1
Desulfuromonas acetoxidans			6.659	0.038							1
Desulfuromonas michiganensis			8.286	0.007							1
Paenibacillus sp. JDR-2			7.741	0.010							1
Pelobacter carbinolicus			7.705	0.011							1
Pelobacter sp. SFB93			7.819	0.010							1
Planctomycetes bacterium Pan216			6.494	0.042							1
Planctomycetes bacterium Pan265			6.608	0.038							1

Pseudomonas sagittaria	7.299	0.017							1
Ruminiclostridium hungatei	7.375	0.045							1
Aridibacter famidurans			7.444	0.038					1
Bacillus cereus			7.079	0.045					1
Candidatus Saccharibacteria bacterium YM_S32_TM7_50_20			7.338	0.038					1
Desulfuromonas soudanensis			7.440	0.036					1
Desulfuromonas sp. DDH964			8.219	0.007					1
Kofleria flava			6.552	0.043					1
Stanieria cyanosphaera			7.400	0.019					1
Dongia mobilis					7.133	0.022			1
Geobacter sp. M21					7.110	0.023			1
Longimicrobium terrae					6.698	0.033			1
Microvirga ossetica					8.043	0.002			1
Aciditerrimonas ferrireducens							4.680	0.032	1
Arthrobacter sp. QXT-31							8.136	0.003	1
Fimbriiglobus ruber							7.274	0.010	1
Geobacter bemidjiensis							6.654	0.035	1
Geobacter uraniireducens							6.968	0.027	1
Lysinibacillus sp. SGAir0095							6.367	0.040	1
Paenibacillus xylanilyticus							6.356	0.040	1
Pedomicrobium americanum							6.935	0.028	1
Rubrobacter radiotolerans							6.587	0.038	1
Solirubrobacter ginsenosidimutans							7.058	0.011	1

Supplementary Table S5. Differential abundance of bacterial taxa with increased abundance in non-autoclaved peach rhizosphere soil as compared to autoclaved peach rhizosphere soil. P-adjusted values which are <0.001 are represented as 0.000. Log2 Fold change was based on log2FC=Log2(non-autoclaved)-Log2(autoclaved) using the DESeq2 package in RStudio.

	**Alfalfa**		**Fescue**		**Corn**		**Tomato**		**No Crop**		
Bacterial Taxa	Log2	Padjust	Log2	Padjust	Log2	Padjust	Log2	Padjust	Log2	Padjust	Count
Arenimicrobium luteum	7.566	0.007	7.800	0.006	7.606	0.001	8.036	0.001	7.588	0.000	5
Bacillus megaterium	5.579	0.003	6.169	0.006	3.762	0.036	5.663	0.001	4.340	0.001	5
Brevitalea aridisoli	9.138	0.000	8.884	0.001	8.627	0.000	9.228	0.000	8.715	0.000	5
Brevitalea deliciosa	10.917	0.000	11.033	0.000	10.587	0.000	10.873	0.000	10.454	0.000	5
Gaiella occulta	9.538	0.000	10.204	0.000	9.710	0.000	10.022	0.000	10.060	0.000	5
Longilinea arvoryzae	5.411	0.029	9.298	0.000	5.299	0.019	8.844	0.000	8.428	0.000	5
Methyloceanibacter caenitepidi	8.097	0.003	8.686	0.000	8.287	0.000	7.710	0.006	8.659	0.000	5
Nitrospira japonica	9.207	0.000	9.193	0.000	5.482	0.035	9.283	0.000	6.201	0.000	5
Peribacillus simplex	5.740	0.015	6.093	0.010	8.624	0.000	4.813	0.011	3.964	0.016	5
Skermanella rosea	8.776	0.000	9.159	0.000	8.647	0.000	9.340	0.000	8.846	0.000	5
Solirubrobacter soli	7.646	0.006	5.113	0.040	8.231	0.000	8.398	0.000	8.355	0.000	5
Desulfuromonas michiganensis	7.551	0.007			7.121	0.009	7.820	0.002	8.397	0.001	4
Gemmata sp. SH-PL17	6.721	0.046			7.548	0.006	8.678	0.000	8.239	0.000	4
Candidatus Saccharibacteria bacterium oral taxon 955	7.170	0.036	7.156	0.012			6.209	0.037	5.370	0.047	4
Skermanella stibiiresistens	9.914	0.000	10.526	0.000			10.408	0.000	10.231	0.000	4
[*Desulfobacterium*] *catecholicum*	9.639	0.000	4.690	0.045	4.159	0.047			8.265	0.001	4
Paenibacillus castaneae	8.491	0.000	8.964	0.000	8.299	0.000	8.970	0.000			4
Bellilinea caldifistulae	7.332	0.009	8.021	0.001	7.019	0.009	7.558	0.000			4
Acidimicrobium ferrooxidans			6.673	0.023	6.117	0.045	6.662	0.021	5.982	0.021	4
Aciditerrimonas ferrireducens			6.228	0.009	6.057	0.008	5.801	0.007	9.624	0.000	4
Actinobacteria bacterium IMCC26256			6.658	0.023	6.126	0.045	7.333	0.004	6.696	0.002	4
Geoalkalibacter subterraneus			8.253	0.000	7.795	0.001	7.606	0.002	8.095	0.000	4
Skermanella aerolata			7.352	0.010	6.816	0.012	7.207	0.004	6.931	0.001	4

Solirubrobacter ginsenosidimutans			7.139	0.012	6.712	0.013	7.208	0.004	7.145	0.000	4
Woeseia oceani			7.274	0.011	6.504	0.035	7.285	0.003	7.593	0.000	4
Pelobacter sp. SFB93	7.415	0.008			7.179	0.009			7.957	0.000	3
Pelobacter carbinolicus	7.110	0.012			6.958	0.010			7.849	0.000	3
Pedomicrobium americanum	6.666	0.022			7.666	0.001			7.536	0.000	3
Thermomarinilinea lacunifontana	7.431	0.027	8.235	0.000					6.619	0.002	3
Candidatus Accumulibacter phosphatis	7.233	0.027	7.786	0.007					7.515	0.001	3
Ornatilinea apprima	5.255	0.039	5.446	0.029					4.104	0.030	3
Bacillus cereus	7.044	0.013	7.490	0.009	7.451	0.007					3
Planifilum fulgidum			6.699	0.023	6.666	0.016			5.701	0.030	3
Skermanella mucosa			7.050	0.013	6.802	0.013			6.960	0.001	3
Bacillus safensis			7.383	0.010	7.453	0.007			6.994	0.001	3
Candidatus Koribacter versatilis			6.752	0.046	6.145	0.044			6.821	0.002	3
Paenibacillus xylanilyticus			7.231	0.011	7.217	0.008	6.860	0.016			3
Candidatus Saccharibacteria bacterium YM_S32_TM7_50_20			7.689	0.007			6.566	0.045	7.042	0.001	3
Desulfomonile tiedjei			6.693	0.048	6.566	0.016			6.928	0.001	3
Paenibacillus prosopidis			7.316	0.010	6.526	0.016			6.890	0.002	3
Paenisporosarcina antarctica			7.176	0.012	7.397	0.001			6.809	0.002	3
[*Brevibacterium*] *frigoritolerans*					4.169	0.020	6.356	0.002	3.712	0.008	3
Candidatus Promineofilum breve					6.318	0.038	7.496	0.002	5.967	0.023	3
Dechlorobacter hydrogenophilus					7.181	0.020	6.961	0.031	6.242	0.006	3
Gemmata massiliana					6.589	0.016	6.560	0.024	5.601	0.035	3
Holophaga foetida					7.465	0.007	6.972	0.016	5.810	0.027	3
Planctomycetes bacterium Pla175					4.420	0.031	4.749	0.029	4.001	0.030	3
Baekduia soli					8.134	0.000	8.545	0.000			2
Terrimonas suqianensis					6.439	0.036	6.733	0.019			2
Pelobacter acetylenicus					8.083	0.000			8.664	0.000	2
Alkalitalea saponilacus					8.066	0.010			6.913	0.016	2
Desulfobulbus propionicus			8.276	0.001	7.582	0.015					2

Arthrobacter sp. KBS0702					7.173	0.009			7.024	0.001	2
Paenibacillus sp. DCT19					7.166	0.009			6.744	0.002	2
Deferrisoma camini					6.829	0.004			5.835	0.048	2
Lutispora thermophila					6.723	0.031			6.513	0.025	2
Rhodoplanes tepidicaeni					6.632	0.016			5.860	0.025	2
Rubrivivax gelatinosus					6.594	0.035			6.117	0.018	2
Pseudolabrys sp. FHR47					6.572	0.035			6.982	0.001	2
Rubrobacter radiotolerans					6.543	0.017			6.662	0.002	2
Bacillus velezensis					6.475	0.036			6.113	0.018	2
Terrimonas pekingensis					6.403	0.037			5.986	0.008	2
Sphingomonas daechungensis					6.329	0.039			6.610	0.003	2
Desulfuromonas acetoxidans					6.312	0.038			6.068	0.019	2
Pseudolabrys taiwanensis					6.282	0.040			6.535	0.009	2
Labilithrix luteola					6.250	0.040			6.408	0.004	2
Terribacillus goriensis					6.124	0.045			5.480	0.040	2
Microvirga sp. 17 mud 1-3					6.110	0.045			5.333	0.049	2
Aquihabitans daechungensis					6.006	0.050			6.566	0.003	2
Desulfuromonas soudanensis					4.998	0.029			9.113	0.000	2
Paenisporosarcina indica	6.897	0.039			7.424	0.007					2
Desulfopila inferna	7.846	0.005	7.310	0.004							2
Desulfotalea psychrophila	7.192	0.029	7.028	0.006							2
Adhaeribacter terreus			6.746	0.048					5.382	0.046	2
Paenisporosarcina macmurdoensis			6.269	0.038					5.632	0.033	2
Vicinamibacter silvestris			3.808	0.019					3.153	0.002	2
Anaeromyxobacter sp. K			8.246	0.000					8.067	0.000	2
Bacillus funiculus							3.191	0.041	2.252	0.035	2
Bacillus pseudomycoides							6.722	0.020	5.968	0.023	2
Conexibacter woesei							7.168	0.004	7.342	0.000	2
Fimbriiglobus ruber							6.701	0.021	6.088	0.007	2

Microlunatus phosphovorus							7.100	0.015	6.444	0.003	2
Paenibacillus xylanexedens							6.706	0.019	6.679	0.002	2
Planctomycetes bacterium ETA_A8							3.836	0.025	3.287	0.049	2
Tepidiforma bonchosmolovskayae							7.501	0.003	3.908	0.032	2
Geobacter psychrophilus	8.344	0.000									1
Haliscomenobacter hydrossis	7.172	0.012									1
Methylobacter luteus	8.624	0.011									1
Methylobacter marinus	7.470	0.031									1
Pseudomonas sagittaria	7.244	0.011									1
Azospira restricta			7.171	0.012							1
Desulfocapsa sulfexigens			6.206	0.045							1
Desulfovirga adipica			6.538	0.035							1
Gallionella capsiferriformans			9.127	0.002							1
Hydrogenophaga taeniospiralis			7.191	0.038							1
Legionella clemsonensis			7.094	0.013							1
Lysinibacillus sp. SGAir0095			6.908	0.040							1
Nitrosospira multiformis			7.991	0.005							1
Oxobacter pfennigii			7.505	0.024							1
Syntrophus aciditrophicus			7.656	0.009							1
Alkalihalobacillus decolorationis					6.090	0.048					1
Azoarcus sp. CIB					6.510	0.037					1
Bacillus altitudinis					6.428	0.037					1
Bacillus thuringiensis					8.054	0.000					1
Maribellus luteus					7.283	0.036					1
Microvirga ossetica					7.375	0.007					1
Microvirga subterranea					6.340	0.038					1
Paenibacillus massiliensis					6.614	0.016					1
Paraflavitalea soli					6.155	0.044					1
Stigmatella aurantiaca					7.122	0.009					1

Gemmata obscuriglobus	6.502	0.026			1
Levilinea saccharolytica	6.940	0.006			1
Paenibacillus guangzhouensis	6.585	0.026			1
Paenibacillus sp. JDR-2	3.746	0.030			1
Paraclostridium bifermentans	7.620	0.009			1
Planctomycetes bacterium ETA_A1	6.286	0.033			1
Pseudarthrobacter sp. NIBRBAC000502772	7.261	0.013			1
Pseudarthrobacter sulfonivorans	7.665	0.008			1
[*Polyangium*] *brachysporum*			6.717	0.002	1
Acidothermus cellulolyticus			6.774	0.002	1
Anaeromyxobacter sp. Fw109-5			7.535	0.003	1
Archangium gephyra			5.978	0.023	1
Aromatoleum aromaticum			6.356	0.013	1
Arthrobacter sp. QXT-31			7.086	0.001	1
Azotobacter chroococcum			7.308	0.001	1
Bacillus halotolerans			5.565	0.019	1
Bacillus pumilus			6.187	0.016	1
Blastococcus saxobsidens			5.560	0.037	1
Burkholderiales bacterium GJ-E10			6.259	0.005	1
Chondromyces crocatus			5.769	0.029	1
Dechloromonas hortensis			3.734	0.046	1
Dechloromonas sp. HYN0024			6.306	0.004	1
Dechlorosoma suillum			5.906	0.024	1
Desulforhopalus singaporensis			6.352	0.015	1
Desulfuromonas sp. DDH964			9.162	0.000	1
Domibacillus indicus			6.440	0.003	1

Geobacter daltonii	7.333	0.001	1
Geobacter metallireducens	6.928	0.002	1
Geobacter pickeringii	8.089	0.000	1
Geobacter sp. FeAm09	6.313	0.006	1
Geobacter sp. M21	6.877	0.002	1
Geobacter uraniireducens	8.426	0.000	1
Haliangium ochraceum	6.006	0.008	1
Hydrogenispora ethanolica	5.357	0.004	1
Hyphomicrobium sulfonivorans	7.134	0.000	1
Ilumatobacter fluminis	7.194	0.000	1
Limnoglobus roseus	5.868	0.011	1
Luteitalea pratensis	2.089	0.044	1
Microlunatus ginsengisoli	5.727	0.030	1
Microvirga brassicacearum	5.338	0.049	1
Microvirga zambiensis	5.334	0.049	1
Minicystis rosea	5.780	0.028	1
Nitrosospira briensis	5.724	0.030	1
Nordella oligomobilis	3.343	0.020	1
Paenibacillus aceris	5.784	0.028	1
Paenibacillus sp. 37	6.578	0.003	1
Pelobacter propionicus	7.329	0.001	1
Planctomycetales bacterium	4.047	0.029	1
Planctomycetes bacterium Pla85_3_4	4.060	0.026	1
Polyangium fumosum	6.007	0.021	1
Rhodoplanes azumiensis	6.466	0.003	1
Rubrobacter xylanophilus	5.545	0.037	1
Shimazuella kribbensis	6.453	0.003	1
Sinorhizobium meliloti	5.752	0.030	1
Sorangium cellulosum	6.727	0.002	1

Sphingomonas lutea	5.731	0.030	1
Stenotrophobacter roseus	5.892	0.024	1
Stenotrophobacter terrae	7.380	0.001	1
Thermanaerothrix daxensis	6.528	0.003	1
Thermoanaerobaculum aquaticum	5.599	0.035	1
Zhizhongheella caldifontis	5.463	0.042	1

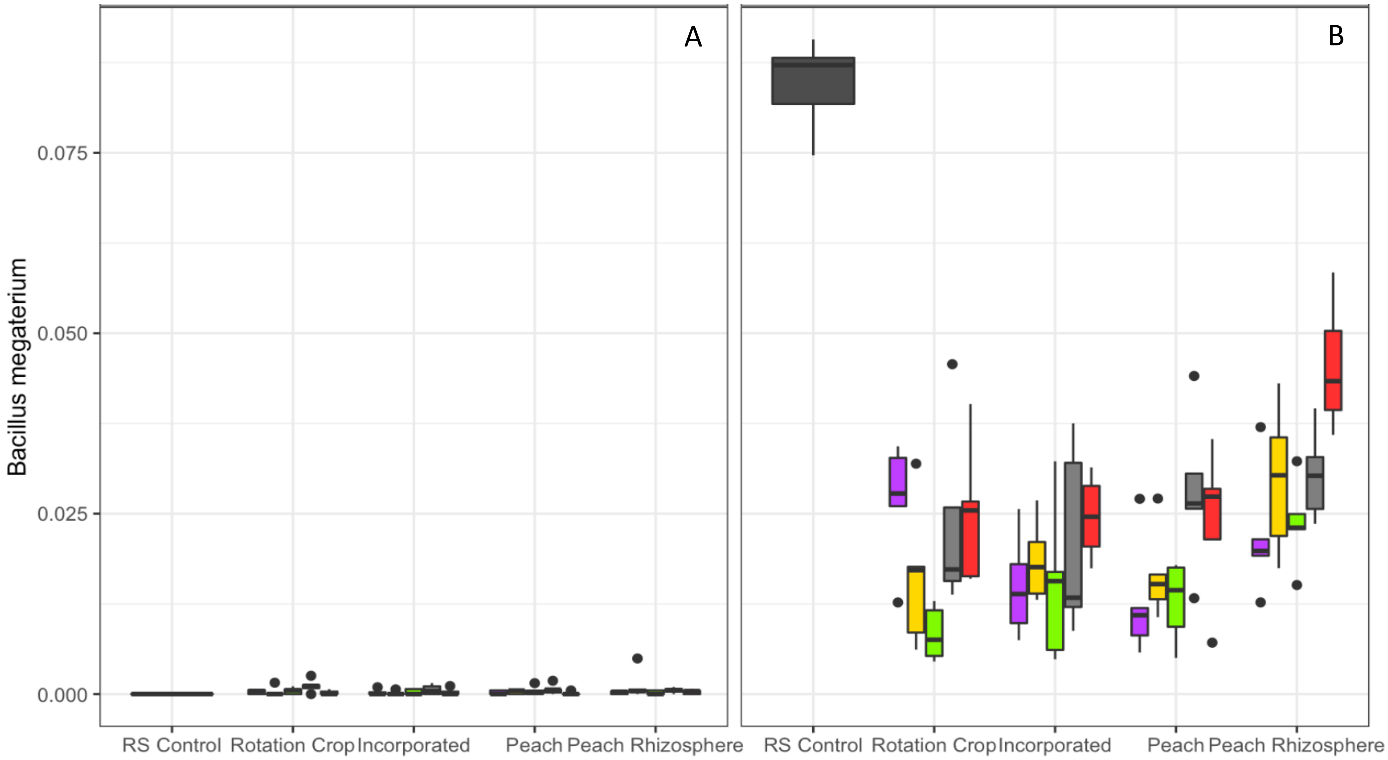

Supplementary Figure S6. *Bacillus megaterium* relative abundance by crop phase. Crop history was denoted by color with alfalfa as purple, fescue as green, corn as yellow, tomato as red, no crop as light grey, and the initial bulk soil control as dark grey. (A)Autoclaved soils. (B) Non-autoclaved soils.
